# Effects of dietary Fibrafid as phytogenic supplementation in standard and nutrient-reduced diets on breast meat quality, carcass traits, histopathology, and feed efficiency in heat-stressed broilers

**DOI:** 10.3389/fvets.2025.1671325

**Published:** 2025-10-10

**Authors:** Maged A. Al-Garadi, Rashed A. Alhotan, Elsayed O. Hussein, Mohammed M. Qaid, Gamaleldin M. Suliman, Mohammed A. Al-Badwi, Esam H. Fazea, Isiaka O. Olarinre

**Affiliations:** Department of Animal Production, College of Food and Agricultural Sciences, King Saud University, Riyadh, Saudi Arabia

**Keywords:** broilers meat quality, feed efficiency, Fibrafid, heat stress, nutrientrequirements

## Abstract

**Introduction:**

Combating heat stress (HS), increasing broiler productivity, and enhancing meat quality are the major priorities in hot climate areas. This study evaluated the effects of Fibrafid, a natural plant-derived product, compared to a commercial prebiotic (TURBO Grow), on meat quality, physicochemical characteristics, carcass features, jejunal histopathology, final average body weight (ABW), and feed conversion ratio (FCR) in heat-stressed broilers fed either a standard or reduced nutrient density diet (diet with a 5% drop in amino acid density and a 1.5% reduction in ME).

**Methods:**

A total of 576 Ross 308 broilers were allocated to eight treatments in a 2 × 4 factorial design, with two diet types (standard vs. reduced) and four additive treatments (none, Fibrafid 0.15%, Fibrafid 0.25%, and TURBO Grow 0.10%). Carcass yield, breast meat physicochemical traits, texture, and intestinal morphology were assessed at 35 days of age, as well as overall ABW and FCR.

**Results and discussion:**

Two-way ANOVA revealed that diet and additive exerted significant main effects on several traits, with some diet × additive interactions. Fibrafid at 0.25% improved water-holding capacity, reduced cooking loss, and increased myofibrillar fragmentation index, while both Fibrafid levels revealed a better gut environment, indicating improved nutrition absorption compared with controls. TURBO Grow supplementation showed intermediate benefits. Carcass weight, carcass yield, and Warner–Bratzler shear force remained unaffected by diet or additives. Reduced diets did not impair breast yield when supplemented with Fibrafid. In conclusion, these results indicate that Fibrafid at 0.25% enhanced meat quality, breast yield, and intestinal integrity in heat-stressed broilers across both dietary regimens, supporting its potential as a functional feed additive under challenging production conditions.

## Highlights

- A 2 × 4 factorial design (diet × additive) tested Fibrafid (0.15 and 0.25%) and TURBO Grow in heat-stressed broilers.
- Fibrafid supplementation enhanced meat quality traits, including water-holding capacity and tenderness.
- At 0.25%, Fibrafid improved meat color stability, lightness, growth performance, and feed efficiency.
- These improvements offer a practical strategy to mitigate production challenges in broilers.
- TURBO Grow supplementation showed intermediate benefits under both standard and nutrient-restricted diets compared to Fibrafid.
- Reduced diets supported comparable carcass yields when combined with Fibrafid, confirming potential for sustainable feeding strategies.
- Histological analysis confirmed improved intestinal integrity and nutrient absorption in supplemented groups.

## 1 Introduction

Poultry production in arid regions such as Saudi Arabia faces significant challenges due to environmental heat stress (HS), which negatively affects broiler growth, carcass yield, and meat quality (1–3). HS leads to oxidative stress (OS), reduced feed intake, and impaired nutrient utilization, causing deteriorations in key meat quality parameters such as water-holding capacity (WHC), pH, tenderness, and color-particularly in the breast muscle, which holds high commercial value (4, 5). These impairments can reduce consumer acceptance and shorten shelf life, ultimately impacting profitability and sustainability in broiler operations.

Among the physicochemical traits used to evaluate meat quality, breast muscle pH is a critical postmortem marker of glycolysis that directly influences WHC, color, and tenderness (6). Meat color, largely determined by myoglobin content and muscle pH, is one of the most influential visual attributes for consumers and signals freshness and quality (7). WHC is also essential, as lean meat contains around 75% water, which can be lost during processing or storage through drip loss (DL), cooking loss (CL), or purge, all of which impact juiciness and yield (8, 9). DL in particular can reflect poor WHC or improper postmortem handling (6). Tenderness, another crucial quality parameter, is often evaluated using the Warner-Bratzler shear force (WBSF) and myofibrillar fragmentation index (MFI). Higher MFI values indicate greater proteolysis and tenderness, while lower WBSF values reflect easier meat cutting and better texture (10, 11). Collectively, these characteristics help define consumer satisfaction, purchasing decisions, and willingness to pay a premium price.

To address HS and OS in poultry, nutritional interventions such as phytogenic feed additives (PFAs) and prebiotics have gained increasing attention. These additives offer antioxidant, anti-inflammatory, and digestive-enhancing properties, which may help maintain performance and meat quality under environmental stress (12–14). At the same time, global concerns over antimicrobial resistance (AMR) have prompted restrictions on antibiotic growth promoters (AGPs), encouraging the poultry industry to adopt safer, more sustainable alternatives (15). Plant-based feed additives enriched with bioactive compounds, including polyphenols, flavonoids, and dietary fibers, are increasingly being used to promote gut health, improve nutrient absorption, and enhance immune responses without relying on AGPs (12, 16, 17).

Fibrafid is a novel, sustainable phytogenic additive formulated from plant extracts and fiber-rich components. Its potential to alleviate OS and enhance nutrient utilization may be particularly beneficial under HS conditions, especially when combined with dietary strategies aimed at reducing feed costs and environmental impact. Notably, reducing dietary metabolizable energy (ME) and amino acid density (AAD) can decrease the carbon footprint and improve feed efficiency. However, such reductions often compromise growth performance unless mitigated by functional additives that maintain digestibility and metabolic support (18).

Given the dual challenges of HS and feed sustainability, this study aimed to assess the combined effect of Fibrafid supplementation and nutrient-reduced diets on broiler performance and meat quality under heat stress. Two diets were formulated: a standard corn–soybean basal diet (positive control, PC1) and a nutrient-reduced diet with 1.5% lower ME and 5% lower AAD (negative control, NC1). Each diet was further supplemented with either Fibrafid at two inclusion levels (0.15% or 0.25%) or a commercial prebiotic (TURBO Grow at 0.10%), resulting in a total of eight treatment groups.

The study focused on evaluating breast meat quality parameters (pH, WHC, DL, CL, WBSF), carcass traits, feed efficiency, and jejunal histopathology in broilers raised under natural cyclic heat stress conditions in Saudi Arabia. By exploring the potential of Fibrafid to counterbalance the limitations of nutrient-reduced diets, the findings aim to provide insights into cost-effective and environmentally sustainable feed strategies that support both production performance and meat quality. Improving feed efficiency and maintaining meat quality under heat stress not only enhances production profitability but also contributes to sustainability and food security, particularly in regions where rising temperatures threaten poultry production systems. By reducing reliance on high-energy diets, such strategies support more resilient poultry production systems in the context of climate change.

## 2 Materials and methods

### 2.1 Experimental design and birds

The experiment was carried out in the poultry experimental unit of the educational and research farm at Al-Amariya, KSU, Riyadh, Saudi Arabia. A total of 576 one-day-old Ross 308 chicks (arrival weight 44.91 ± 0.11 g) were obtained from Al Khumasia Company and randomly allocated to 48-floor pens, with 12 birds per pen. The dietary treatments were arranged in a 2 × 4 factorial arrangement, with two diet types (standard vs. reduced nutrient density) and four additive treatments (none, Fibrafid at 0.15%, Fibrafid at 0.25%, and TURBO Grow at 0.10%).

This resulted in eight experimental groups:

1. Standard diet without additives “Positive control diet (PC1)”: Met nutrient requirements of Ross 308 based on Ross Nutrient Specifications—All plant protein based feeds—Aviagen 2022 handbook.
2. Standard diet + 0.15% Fibrafid (PC1 + 1.5 kg Fibrafid/ton).
3. Standard diet + 0.25% Fibrafid (PC1 + 2.5 kg Fibrafid/ton).
4. Standard diet + 0.10% TURBO Grow (PC1 + 0.10% TURBO Grow; “PC2”).
5. Reduced diet without additives: Negative control diet (NC1): Diet with −5% amino acid density and −1.5% ME.
6. Reduced diet + Fibrafid 0.15%: (NC1 + 1.5 kg Fibrafid/ton).
7. Reduced diet + Fibrafid 0.25%: (NC1 + 2.5 kg Fibrafid/ton).
8. Reduced diet + 0.10% TURBO Grow: (NC1 + 0.10% TURBO Grow; “NC2”).

Each treatment was replicated in six replicated pens of equal size (each pen 1 m^2^), with equal numbers of males and females per pen (6♂ & 6♀). Birds were raised to 35 days of age under standard husbandry conditions with *ad libitum* access to feed and water. The feeders and waterers were adjusted according to the chicks’ growth stages.

### 2.2 Diets and feed additives

The standard diet was formulated according to the Aviagen Ross 308 nutrient specifications (19), while the reduced diet contained lower metabolizable energy (ME) and amino acid levels (approximately 1.5% less ME and 5% less amino acid density (AAD)). Diets were provided in mashed form for the starter, grower, and finisher periods (0–9, 10–23, and 24–35 days, respectively), with variations in ME and AAD detailed in Supplementary Table S1. The nutritive composition of the basal diet was analyzed using standardized methods AACC (20) and utilized to formulate diets.

Fibrafid was included at 0.15% or 0.25% of the diet, while TURBO Grow was provided at 0.10%. Fibrafid® is a natural product extracted from plants, was produced by Phytaxis SA, a Swiss company, and contains 83% wheat straw and munj Sweetcane hydrolyzed fiber, 6% glycerol, 5% lactic acid, 5% citric acid, and 1% benzoic acid. According to manufacturer information, Turbo Grow® powder is a commercial prebiotic blend of mannan-oligosaccharides (MOS), betaine, yeast extract, citric acid, propionic acid, formic acid, and diatomaceous earth at levels of 50,000, 60,000, 385,000, 20,000, 38,500, 21,000, and 100,000 mg/kg, respectively.

### 2.3 Microclimate and housing

Chicks were housed in floor pens with wood shavings as litter at a stocking density of 12 birds/m^2^, each pen = 1 m^2^. A continuous fluorescent light program was used 24 h/day to minimize circadian variation during the trail. The brooding temperature was set at 33 ± 1 °C on arrival and lowered daily until reaching 24 ± 1 °C on day 23. Heat stress was applied from day 24 to 35 by increasing the room temperature to 34 ± 1 °C for 8 h/day from 08:00 to 16:00 and 22 ± 1 °C for the rest of the time per day, and relative humidity (RH) was around 50 to 60%. During the experiment, the average outdoor temperature was 35 ± 4 °C, and the RH was 31 ± 5 °C. Birds were vaccinated at hatch against Newcastle disease and infectious bursal disease following Ross 308 guidelines (19).

### 2.4 Carcass traits and meat quality measurements

At the end of the study (day 35), one bird per replicate (six birds per treatment) with a body weight close to the group mean was randomly selected for sampling. Sampling was balanced by sex, with equal numbers of males and females included. The birds fasted for 12 h prior to slaughter, with only water provided. Individual birds were weighed and manually slaughtered by cutting the jugular vein under standard conditions. After scalding in a 50–60 °C water tank for 1–1.5 min, the birds were plucked, chilled in cold water, and processed. The hot carcass, breast, leg quarters, back-wings-neck, abdominal fat pad, liver, heart, proventriculus, pancreas, and lymphoid organs were weighed separately to determine percentage yields of each component (g/100 g hot carcass weight) (21).

Physicochemical parameters, WHC%, DL%, CL%, WBSF, and TPA were evaluated for meat quality.

#### 2.4.1 Physicochemical parameters

Breast pH, internal temperature, and color quality (lightness L*, redness a*, and yellowness b*) were measured on the cranial part of the breast fillet in duplicate 24 h postmortem (PM_24_). To assess postmortem glycolysis and meat quality and to monitor post-slaughter chilling efficiency and ensure food safety, a pH meter probe with thermocouple (model pH 211, Woonsocket, RI, USA) was used. To evaluate visual appeal and consumer preference, a Minolta CR-400 chromameter (Konica Minolta, Tokyo, Japan) was used. To provide a far more precise estimate of how customers perceive the color of meat, additional color derivatives, such as ∆E*, a*:b* ratio, chroma, hue angle (H°), whiteness index, and browning index, were calculated (22, 23).

#### 2.4.2 WHC, CL, and DL

To measure the ability of meat to retain water during processing and storage, WHC was determined by pressing 2 g of breast fillet with a 10 kg weight for 5 min, calculating the difference between pre-pressed and final pressed weights as described by Wilhelm, Maganhini (24). To determine the amount of water and fat lost during cooking, CL% was calculated by grilling fillets on a tabletop grill (Kalorik GR 28,215; Kalorik, Miami Gardens, FL, USA) until their internal temperature reached 70 °C, recording the weight difference before and after cooking multiplied by 100 (25). To measure the release of water from meat during storage, DL% was assessed by suspending meat samples for 24 h at refrigeration temperature and recording weight loss due to dripping.

#### 2.4.3 MFI, tenderness, and mechanical properties of meat

To assess the degree of muscle fiber breakdown, MFI was determined spectrophotometrically (Hach, Loveland, CO, USA) at 540 nm after homogenizing minced samples in MFI buffer (26). To objectively measure meat tenderness and to evaluate the mechanical properties of meat, such as hardness, springiness, chewiness, and cohesiveness, WBSF and TPA were measured using a TA-HD Texture Analyzer (Stable Micro Systems Ltd., Godalming, UK) with a Warner-Bratzler blade and cylindrical compression piston, respectively. Five rectangular core steaks per fillet were subjected to WBSF and TPA tests under standardized conditions (23, 27).

### 2.5 Average targeted body weight and feed conversion ratio

Average body weight (ABW) and feed intake (FI) were measured at the beginning and end of each period. Feed conversion ratio (FCR) was computed as FI divided by body weight gain (BWG). Mortality was recorded daily, and any associated data were accounted for in the analyses.

### 2.6 Jejunal histopathological changes

Jejunal samples were collected and fixed in 10% formalin. Sections were embedded in paraffin, cut at 5 μm thickness, and stained with hematoxylin and eosin (H&E). Villus and mucosal integrity were examined under a light microscope to evaluate gut health.

### 2.7 Statistical analysis

Data were analyzed using a two-way ANOVA with diet (standard vs. reduced) and additive (none, Fibrafid 0.15%, Fibrafid 0.25, and 0.10% TURBO Grow) as the main factors and their interaction in the Institute’s Statistical Analysis System 9.4 SAS (28) software. Prior to analysis, data were tested for normality (Shapiro–Wilk) and homogeneity of variances (Levene’s test). When significant effects were detected, means were separated using Tukey’s post-hoc multiple comparison test. Pen was considered the experimental unit for performance data, while the individual bird was the unit for carcass and meat quality. Statistical significance was set at *p* < 0.05, and results are illustrated as means ± standard error of the mean (SEM).

## 3 Results

### 3.1 Physicochemical properties

The physicochemical parameters (core temperatures, pH, and color components and it derivatives) at PM_24_ of the 35-day-old bird breast samples are shown in Tables 1 and 2. As presented in Table 1, core temperature of breast muscle was influenced by diet (*p* = 0.013), with reduced diets showing slightly (*p* < 0.0001) lower values. Additive effects were also evident, with Fibrafid at 0.15% yielding the highest core temperature. The pH of breast muscle was influenced by diet (*p* = 0.001), with reduced diets showing slightly higher values. Muscle pH remained within the normal physiological range (5.61–5.69) and was not altered (*p* > 0.05) by additive supplementation. No significant diet by additive interaction was observed (*p* > 0.05) on core temperature and pH of breast muscle.

**Table 1:** Effects of diet, additive, and their interaction on physiochemical values at 24 h post-mortem of breast meat of broilers slaughtered at day 35 of age.

Treatments		Core temperature	pH	Color component		
Diet	Additive			Lightness (L*)	Redness (a*)	Yellowness (b*)
Interaction effects						
Standard	None	17.15	5.64	51.2^ab^	1.12^b^	13.18^ab^
Standard	Fibrafid 0.15%	18.23	5.61	48.41^b^	2.59^a^	13.06^ab^
Standard	Fibrafid 0.25%	17.93	5.65	50.73^ab^	0.56^c^	11.90^c^
Standard	TURBO Grow	17.46	5.61	52.96^ab^	0.05^d^	13.95^a^
Reduced	None	17.06	5.68	52.22^ab^	0.96^b^	14.03^a^
Reduced	Fibrafid 0.15%	17.97	5.69	53.03^a^	−0.64^e^	12.88^ab^
Reduced	Fibrafid 0.25%	17.59	5.69	53.34^a^	−0.15^d^	13.32^ab^
Reduced	TURBO Grow	17.09	5.69	53.05^a^	0.59^c^	14.02^a^
SEM		0.079	0.009	0.405	0.137	0.122
Main effects of diet						
Standard		17.69^a^	5.63^b^	50.83^b^	1.08^a^	13.03^b^
Reduced		17.43^b^	5.69^a^	52.91^a^	0.19^b^	13.57^a^
SEM		0.112	0.012	0.573	0.193	0.173
Main effects of additive						
None		17.11^c^	5.66	51.71	1.04^a^	13.61^a^
Fibrafid 0.15%		18.10^a^	5.65	50.72	0.98^a^	12.97^b^
Fibrafid 0.25%		17.76^b^	5.67	52.03	0.21^b^	12.61^b^
TURBO Grow		17.28^c^	5.65	53.01	0.33^b^	13.99^a^
SEM		0.159	0.017	0.811	0.274	0.244
*p* value						
Diet		0.013	0.001	0.008	<0.0001	0.001
Additive		<0.0001	0.754	0.208	<0.0001	<0.0001
Diet*Additive		0.712	0.735	0.020	<0.0001	0.004

*n* = 6 per each additive*diet arrangement. ^a–e^Means in the same column with different superscripts differ significantly (*p* < 0.05). Means with the same letter are not significantly different.

**Table 2:** Effects of diet, additive, and their interaction on color derivatives values at 24 h post-mortem of breast meat of broilers slaughtered at day 35 of age.

Treatments		Color derivatives					
Diet	Additive	Delta E	Chroma	Hue angle	BI	WI	a/b ratio
Interaction effects							
Standard	None	43.97^ab^	13.23^ab^	85.15^a^	30.8^bc^	49.43^ab^	0.085^b^
Standard	Fibrafid 0.15%	46.72^a^	13.32^ab^	78.75^a^	35.03^a^	46.71^b^	0.199^a^
Standard	Fibrafid 0.25%	44.17^ab^	11.92^c^	87.32^a^	27.07^cd^	49.3^ab^	0.047^c^
Standard	TURBO Grow	42.42^ab^	13.95^a^	−0.20^ab^	30.01^bcd^	50.92^ab^	0.004^d^
Reduced	None	43.16^ab^	14.06^a^	86.07^a^	31.96^ab^	50.19^ab^	0.069^bc^
Reduced	Fibrafid 0.15%	42.13^b^	12.9^bc^	−87.13^b^	26.29^d^	51.28^a^	−0.05^e^
Reduced	Fibrafid 0.25%	41.91^b^	13.32^ab^	0.67^ab^	27.86^bcd^	51.47^a^	−0.012^d^
Reduced	TURBO Grow	42.35^ab^	14.04^a^	87.57^a^	30.86^bc^	50.99^ab^	0.042^c^
SEM		0.391	0.121	10.976	0.492	0.385	0.010
Main effects of diet							
Standard		44.32^a^	13.10^b^	62.75^a^	30.73^a^	49.09^b^	0.08^a^
Reduced		42.39^b^	13.58^a^	21.79^b^	29.24^b^	50.98^a^	0.01^b^
SEM		0.553	0.172	15.522	0.695	0.545	0.015
Main effects of additive							
None		43.56	13.65^a^	85.61^a^	31.38^a^	49.81	0.076^a^
Fibrafid 0.15%		44.43	13.11^b^	−4.19^c^	30.66^a^	48.99	0.073^a^
Fibrafid 0.25%		43.04	12.62^c^	43.99^b^	27.46^b^	50.39	0.018^b^
TURBO Grow		42.39	13.99^a^	43.68^b^	30.43^a^	50.95	0.022^b^
SEM		0.782	0.243	21.952	0.983	0.770	0.021
*p* value							
Diet		0.011	0.004	0.007	0.034	0.012	<0.0001
Additive		0.255	<0.0001	0.001	0.001	0.267	<0.0001
Diet*Additive		0.027	0.001	<0.0001	<0.0001	0.028	<0.0001

Color derivative: Delta E (∆E): total color change; Chroma: Saturation index; BI: browning index; WI: whiteness index; a*/b*: redness/yellowness ratio. *n* = 6 per each additive*diet arrangement. ^a–e^Means in the same column with different superscripts differ significantly (*p* < 0.05). Means with the same letter are not significantly different.

Lightness (L*), redness (a*), and yellowness (b*) were affected by diet, additive, and their interactions, except for lightness not affected by additives. Notably, reduced diets combined with Fibrafid (0.25%) or TURBO Grow increased lightness, whereas redness was highest with Fibrafid 0.15% under the standard diet. These results indicate that both diet and additive influenced breast meat color parameters. Total color difference (∆E), Chroma (saturation index), and Hue angle were influenced by both diet and additive, with several strong diet × additive interactions (*p* < 0.001; Table 2). Birds on reduced diets had lower ∆E but higher Chroma values than those on standard diets. Additives differentially affected color stability, with Fibrafid at 0.25% reducing Chroma and with Fibrafid at 0.15% reducing Hue angle compared with controls, while TURBO Grow enhanced Chroma values. The browning index (BI) of breast muscle was significantly lower in reduced diets, (*p* = 0.034), with Fibrafid at 0.15% producing the lowest values (*p* < 0.0001). The whiteness index (WI) was significantly higher in reduced diets, with Fibrafid at 0.25 and 0.15% producing the highest. While the a*/b* ratio was strongly affected by additive type, with Fibrafid at 0.15% producing the highest values particularly under the standard diet.

### 3.2 Meat quality and texture profile analysis

The meat quality characteristics (WHC%, DL%, CL%, MFI, WBSF and TPA) for the bird samples at 35 days of age are shown in Tables 3 and 4. As shown in Table 3, CL% and MFI were significantly affected by diet type (*p* < 0.001), with reduced diet were the highest CL% and lowest MFI values compared to standard diet. DL and WHC% were not significantly affected by diet but were influenced by additive supplementation (*p* < 0.001). Meat quality parameters were significantly affected by additive supplementation (*p* < 0.001), with the highest WHC%, lowest CL%, and strongest MFI in Fibrafid-supplemented group at 0.25% inclusion. However, WBSF did not differ significantly among treatments or their interactions (*p* > 0.05), confirming that tenderness improvements were due mainly to structural changes (MFI and texture profile parameters) rather than shear force reductions.

**Table 3:** Effects of diet, additive, and their interaction on meat quality parameters in breast meat of broilers at 35 days of age.

Treatments		Meat quality				
Diet	Additive	WHC%	DL%	CL%	MFI	WBSF (N/cm^2^)
Interaction effects						
Standard	None	65.26^abc^	5.27^bc^	29.66^b^	54.6^d^	3.10
Standard	Fibrafid 0.15%	67.2^ab^	5.00^bc^	25.63^d^	58.02^ab^	2.96
Standard	Fibrafid 0.25%	67.98^a^	5.54^ab^	22.11^f^	59.45^a^	2.96
Standard	TURBO Grow	67.0^ab^	5.12^bc^	25.88^d^	55.2^d^	3.22
Reduced	None	63.17^c^	6.15^a^	31.51^a^	51.22^e^	3.09
Reduced	Fibrafid 0.15%	67.41^ab^	4.99^bc^	27.87^c^	57.3^bc^	2.95
Reduced	Fibrafid 0.25%	68.03^a^	4.74^c^	23.77^e^	59.04^a^	2.90
Reduced	TURBO Grow	64.33^bc^	5.57^ab^	26.09^d^	56.18^cd^	3.03
SEM		0.358	0.083	0.423	0.389	0.039
Main effects of diet						
Standard		66.86	5.23	25.82^b^	56.82^a^	3.06
Reduced		65.74	5.36	27.31^a^	55.93^b^	2.99
SEM		0.506	0.117	0.599	0.550	0.055
Main effects of additive						
None		64.22^c^	5.71^a^	30.59^a^	52.91^d^	3.10
Fibrafid 0.15%		67.30^ab^	4.99^c^	26.75^b^	57.66^b^	2.95
Fibrafid 0.25%		68.00^a^	5.14^bc^	22.94^d^	59.25^a^	2.93
TURBO Grow		65.67^b^	5.34^ab^	25.98^c^	55.69^c^	3.13
SEM		0.716	0.165	0.847	0.777	0.077
*p* value						
Diet		0.074	0.197	<0.0001	0.002	0.506
Additive		0.0001	0.0007	<0.0001	<0.0001	0.130
Diet*Additive		0.0003	<0.0001	0.0023	<0.0001	0.948

WHC, Water holding capacity; DL, Dripping loss; CL, Cooking loss; MFI, Myofibril fragmentation index, and WBSF, Warner-Bratzler shear force. *n* = 6 per each additive*diet arrangement. ^a–c^Means in the same column with different superscripts differ significantly (*p* < 0.05). Means with the same letter are not significantly different.

**Table 4:** Effects of diet, additive, and their interaction on texture profile analysis (TPA) in breast meat of broilers at 35 days of age.

Treatments		TPA				
Diet	Additive	Hardness	Springiness	Cohesiveness	Chewiness	Gumminess
Interaction effects						
Standard	None	7.37^c^	0.671^bc^	0.455^cd^	2.36^ab^	3.35
Standard	Fibrafid 0.15%	6.94^d^	0.758^b^	0.50^b^	2.33^b^	3.47
Standard	Fibrafid 0.25%	6.22^e^	0.801^a^	0.554^a^	2.08^c^	3.45
Standard	TURBO Grow	6.80^d^	0.708^b^	0.47^bc^	2.16^bc^	3.20
Reduced	None	8.50^a^	0.636^c^	0.448^cd^	2.57^a^	3.81
Reduced	Fibrafid 0.15%	8.03^b^	0.632^c^	0.43^d^	2.16^bc^	3.46
Reduced	Fibrafid 0.25%	7.93^b^	0.686^bc^	0.45^cd^	2.25^bc^	3.57
Reduced	TURBO Grow	7.98^b^	0.685^c^	0.434^d^	2.57^a^	3.46
SEM		0.109	0.008	0.007	0.030	0.039
Main effects of diet						
Standard		6.83^b^	0.735^a^	0.495^a^	2.23^b^	3.37^b^
Reduced		8.11^a^	0.661^b^	0.440^b^	2.39^a^	3.57^a^
SEM		0.154	0.012	0.010	0.043	0.056
Main effects of additive						
None		7.93^a^	0.655^c^	0.450^b^	2.46^a^	3.58
Fibrafid 0.15%		7.48^b^	0.697^b^	0.465^b^	2.25^bc^	3.46
Fibrafid 0.25%		7.07^c^	0.743^a^	0.502^a^	2.17^c^	3.51
TURBO Grow		7.39^b^	0.696^b^	0.452^b^	2.37^b^	3.33
SEM		0.217	0.017	0.014	0.060	0.079
*p* value						
Diet		<0.0001	<0.0001	<0.0001	0.0001	0.005
Additive		<0.0001	<0.0001	0.0002	<0.0001	0.103
Diet*Additive		0.002	<0.0001	0.0008	<0.0001	0.118

*n* = 6 per each additive*diet arrangement. ^a–c^ Means in the same column with different superscripts differ significantly (*p* < 0.05). Means with the same letter are not significantly different.

Significant diet × additive interactions were observed for meat quality traits, except for WBSF, indicating that additive effects varied depending on diet type. Birds supplemented with Fibrafid at 0.25% under both standard and reduced diets exhibited the highest (*p* < 0.05) WHC% and MFI%, alongside the lowest DL% and CL%, compared with the other treatments. These findings indicate that Fibrafid at 0.25% enhanced meat quality regardless of diet, as evidenced by improved WHC, greater proteolysis (higher MFI), and reduced water and cooking losses. In contrast, the negative control (Reduced diet without any un-supplementation) consistently showed the poorest performance across these parameters.

As shown in Table 4, texture profile attributes were significantly (*p* < 0.05) affected by both diet and additive. Birds on the reduced diet had higher hardness, chewiness, and gumminess but lower springiness and cohesiveness compared with those on the standard diet. Additive supplementation, particularly Fibrafid at 0.25%, reduced hardness and chewiness and improved springiness and cohesiveness, indicating a more favorable texture. Significant diet × additive interactions on TPA attributes except for gumminess, were observed. Fibrafid at 0.25% under the standard diet produced the highest (p < 0.05) springiness and cohesiveness, while hardness and chewiness were significantly reduced compared with the other groups, indicating that Fibrafid, particularly at 0.25%, improved textural properties more effectively in standard diets than in reduced diets. Collectively, these results demonstrate that Fibrafid at 0.25% produced the most favorable overall meat quality profile, whereas the reduced diet without any un-supplementation group exhibited the least desirable outcome.

### 3.3 Carcass traits

The carcass characteristics of 35-day-old broilers treated feed with or without Fibrafid feed are shown in Tables 5, 6. As shown in Table 5, slaughter weight and carcass weight were not significantly affected by diet or additive, although reduced diets produced slightly higher carcass weights. Breast yield was significantly increased in reduced diets and was further enhanced by Fibrafid supplementation compared with controls. No significant differences (*p* > 0.05) were observed for leg, back-wing-neck, or internal organ percentages, except for the gizzard, which was affected by the additive and by the diet*additive interaction. Also, a significant diet × additive interaction (*p* = 0.008) was found for breast yield, where Fibrafid supplementation increased breast proportion in both reduced diets and standard diets, followed by TURBO Grow in reduced diet. Dressing yield remained unaffected across treatments.

**Table 5:** Effects of diet, additive, and their interaction on carcass characteristics of broilers at 35 days of age.

Treatments		Live weight (g)	Carcass weight (g)	Dressing yield (%)	% Carcass weight		
Diet	Additive				Breast	Legs	Back-Wings-Neck
Interaction effects							
Standard	None	2,638	1924	72.9	34.9^b^	39.8	25.3
Standard	Fibrafid 0.15%	2,621	1958	74.7	36.8^a^	37.6	25.6
Standard	Fibrafid 0.25%	2,687	2003	74.5	36.8^a^	40.2	23.0
Standard	TURBO Grow	2,621	1898	72.4	33.8^b^	39.7	26.5
Reduced	None	2,782	1996	71.8	35.9^ab^	37.3	24.7
Reduced	Fibrafid 0.15%	2,750	2039	74.1	36.6^a^	38.9	24.5
Reduced	Fibrafid 0.25%	2,721	2037	74.9	36.5^a^	39.3	24.7
Reduced	TURBO Grow	2,629	1992	75.8	36.1^a^	38.5	24.4
SEM		27.05	20.12	0.40	0.302	0.302	0.320
Main effects of diet							
Standard		2,642	1946	73.6	35.6^b^	39.3	25.1
Reduced		2,720	2016	74.1	36.9^a^	38.5	24.6
SEM		38.3	28.5	0.571	0.427	0.427	0.452
Main effects of additive							
None		2,710	1960	72.4	36.4	38.5	25.0
Fibrafid 0.15%		2,686	1998	74.4	36.7	38.3	25.0
Fibrafid 0.25%		2,704	2020	74.7	36.4	39.8	23.8
TURBO Grow		2,625	1945	74.1	35.4	39.1	25.4
SEM		54.10	40.235	0.808	0.603	0.604	0.640
*p* value							
Diet		0.165	0.094	0.472	0.014	0.151	0.422
Additive		0.696	0.550	0.1476	0.359	0.266	0.312
Diet*Additive		0.714	0.616	0.147	0.008	0.132	0.274

*n* = 6 per each additive*diet arrangement. ^a,b^Means in the same column with different superscripts differ significantly (*p* < 0.05). Means with the same letter are not significantly different.

**Table 6:** Effects of diet, additive, and their interaction on an internal organs of broilers carcass at 35 days of age.

Treatments		% Carcass weight								
Diet	Additive	Heart	Liver	Prov.	Gizzard	Bursa	Spleen	Thymus	AF	Pancreas
Interaction effects										
Standard	None	0.623	3.01	0.699	1.82^ab^	0.207	0.143	0.285^bc^	1.23	0.231
Standard	Fibrafid 0.15%	0.586	2.49	0.676	1.81^ab^	0.224	0.137	0.386^a^	1.23	0.245
Standard	Fibrafid 0.25%	0.609	2.61	0.632	1.97^a^	0.251	0.12	0.298^abc^	1.06	0.241
Standard	TURBO Grow	0.613	2.71	0.716	1.88^ab^	0.288	0.133	0.315^abc^	1.05	0.257
Reduced	None	0.674	2.51	0.562	1.73^b^	0.247	0.131	0.287^bc^	1.03	0.233
Reduced	Fibrafid 0.15%	0.654	2.75	0.617	2.01^a^	0.255	0.139	0.279^c^	1.23	0.235
Reduced	Fibrafid 0.25%	0.646	2.47	0.667	2.22^a^	0.262	0.13	0.379^ab^	1.22	0.248
Reduced	TURBO Grow	0.622	2.73	0.714	1.84^ab^	0.246	0.108	0.324^abc^	1.13	0.23
SEM		0.011	0.047	0.023	0.036	0.006	0.005	0.009	0.043	0.006
Main effects of diet										
Standard		0.61	2.71	0.68	1.87	0.24	0.14	0.32	1.14	0.245
Reduced		0.65	2.61	0.64	1.95	0.25	0.13	0.31	1.23	0.237
SEM		0.015	0.066	0.033	0.052	0.009	0.006	0.013	0.060	0.008
Main effects of additive										
None		0.65	2.76	0.63	1.78^b^	0.23	0.14	0.29	1.13	0.233
Fibrafid 0.15%		0.62	2.62	0.65	1.91^b^	0.24	0.14	0.32	1.35	0.240
Fibrafid 0.25%		0.63	2.54	0.65	2.10^a^	0.26	0.12	0.34	1.18	0.246
TURBO Grow		0.62	2.72	0.72	1.86^b^	0.27	0.14	0.32	1.09	0.245
SEM		0.022	0.093	0.047	0.073	0.012	0.009	0.018	0.085	0.011
*p* value										
*Diet*		0.063	0.289	0.389	0.230	0.357	0.118	0.583	0.303	0.538
*Additive*		0.708	0.263	0.613	0.009	0.093	0.128	0.097	0.141	0.856
Diet*Additive		0.826	0.051	0.616	0.017	0.086	0.064	0.0003	0.194	0.704

AF, Abdominal fat; Prov, Proventriculus. *n* = 6 per each additive*diet arrangement. ^a–c^Means in the same column with different superscripts differ significantly (*p* < 0.05). Means with the same letter are not significantly different.

As shown in Table 6 relative weights of internal organs were not significantly affected by diet or additive. However, gizzard weight was significantly increased by Fibrafid 0.25% compared with other groups, and thymus lymphoid organ weight was also higher in Fibrafid-supplemented groups, particularly under standard diets (*p* < 0.001 for diet × additive). The spleen and bursa did not differ significantly across treatments.

### 3.4 Target body weight and feed conversion ratio

**Figure 1:**
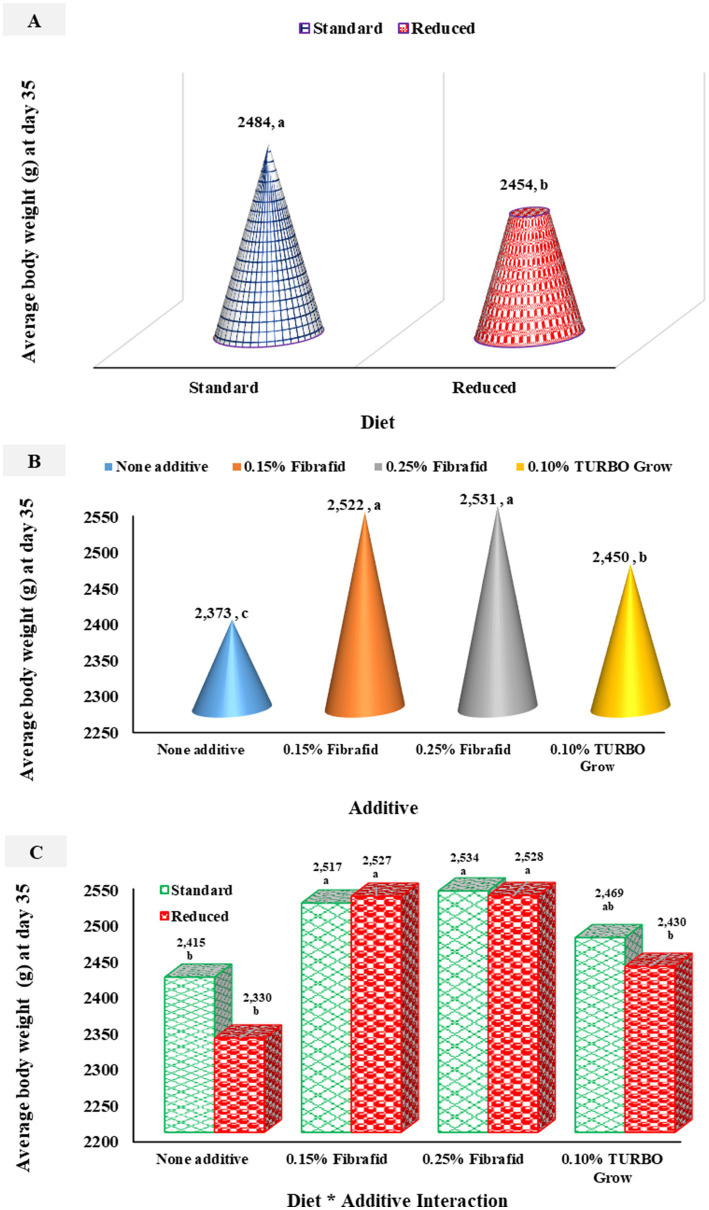
Effects of diet **(A)**, additive **(B)**, and their interaction **(C)** on average body weight. *n* = 6 replicated cage per treatment. ^a–c^Means with different superscripts differ significantly (*p* < 0.05). SEM = 15.72, 22.23, 11.12 for diet, additive, and their interaction, respectively.

As shown in Figures 1A–C, average body weight (ABW) was significantly (*p* < 0.05) influenced by diet, additive, and their interactions. Birds fed the standard diet tended to be heavier than those on the reduced diet (*p* = 0.008). Additive supplementation also improved ABW (*p* < 0.0001) compared with unsupplemented controls (Figure 1A), with Fibrafid at 0.25 and 0.15% producing the highest weights, followed by TURBO Grow (Figure 1B). A significant diet × additive interaction was observed (*p* = 0.021) for ABW, where birds supplemented with Fibrafid at 0.25 and 0.15% achieved the highest weights under both standard and reduced diets (Figure 1C). This was followed by birds receiving TURBO Grow under the standard diet. These results indicate that Fibrafid exerted a consistently positive effect on growth performance across both dietary regimens.

**Figure 2:**
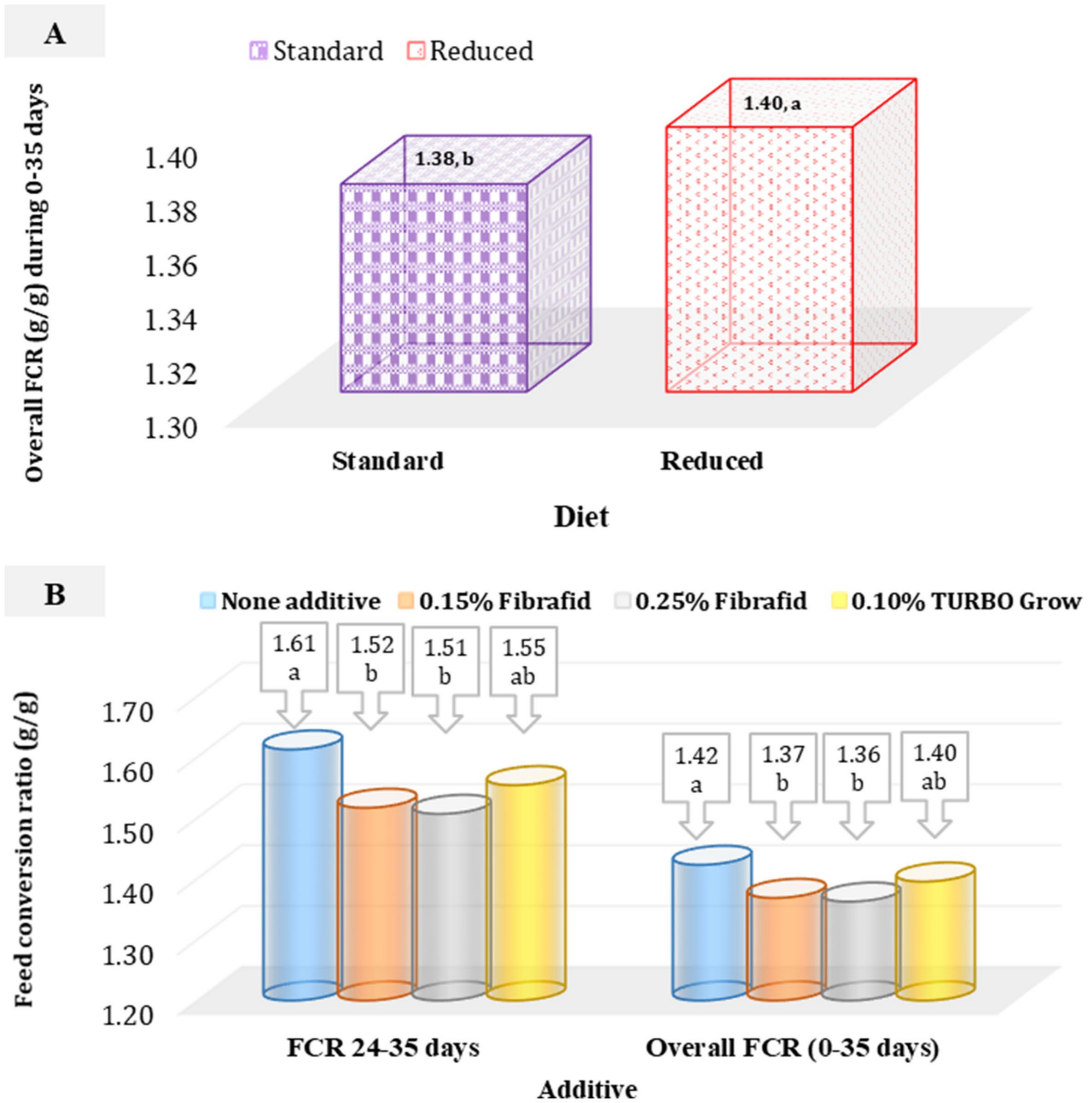
**(A)** Effect of diet on overall feed conversion ratio (FCR) of heat-stressed broilers (SEM = 0.009). **(B)** Effect of additive on FCR of heat-stressed broilers at 24–35 days (SEM = 0.023), and overall period (SEM = 0.012). *n* = 6 replicated cage per treatment. ^a,b^Means with different superscripts differ significantly (*p* < 0.05).

Feed conversion ratio (FCR) along dietary experiment was significantly affected by both diet and additive (Figures 2A,B). Standard diets (diet that meets the requirements of broilers) improved FCR overall periods (0–35 days) compared with reduced diets (diet with a 5% lower amino acid density and 1.5% lower ME value; Figure 2A), while Fibrafid at 0.25 and 0.15% during stressed period (24–35 days) and overall period (0–35 days) improved efficiency relative to the control, and TURBO Grow was intermediate (Figure 2B). However, no significant interaction was detected (*p* > 0.05), confirming that responses to supplementation were independent of diet type.

### 3.5 Jejunal histopathological changes

**Figure 3:**
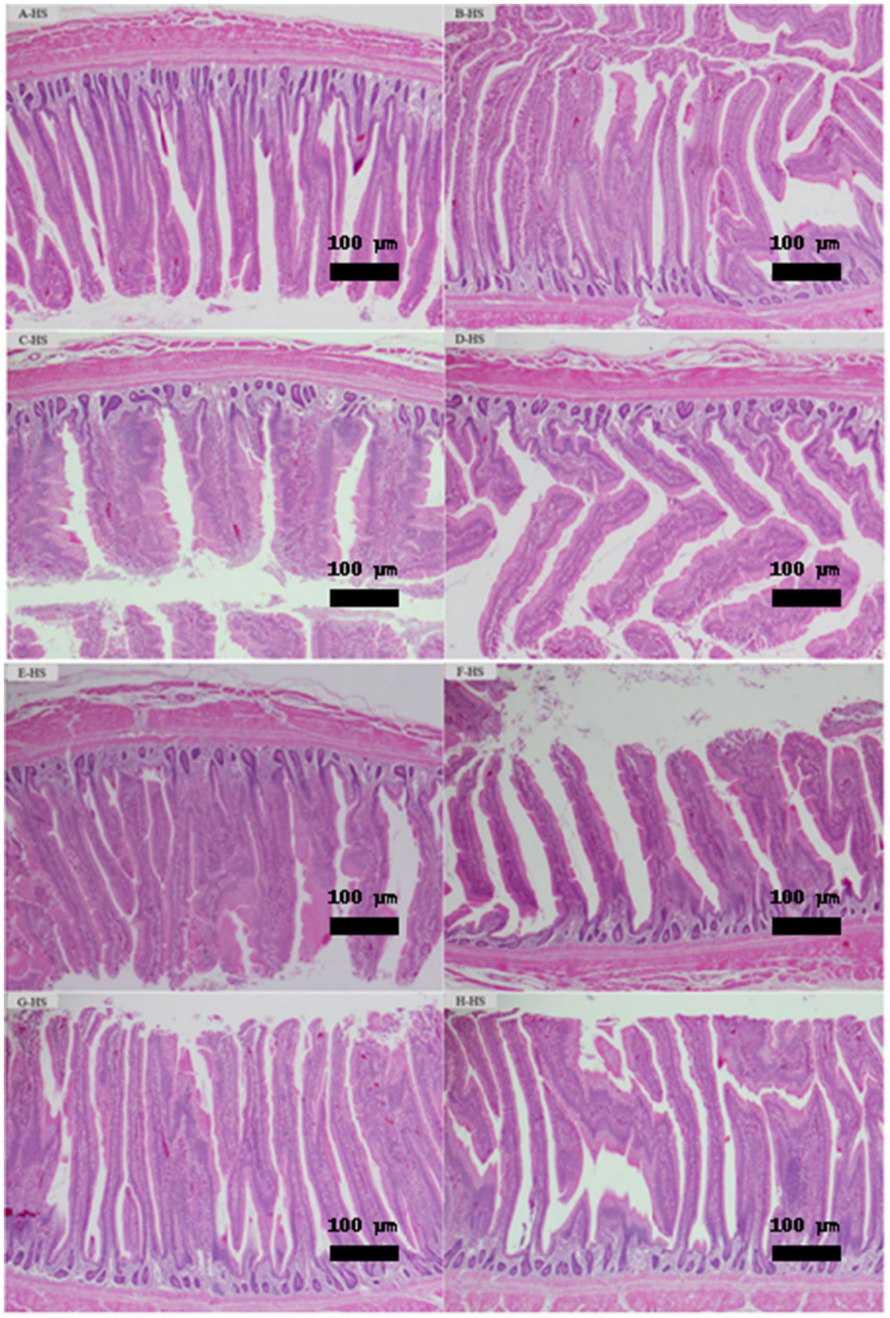
**(A–H)** Treatments: **(A)** Standard diet without additives; **(B)** Standard diet + 0.15% Fibrafid; **(C)** Standard diet + 0.25% Fibrafid; **(D)** Standard diet + 0.10% TURBO Grow; **(E)** Reduced diet without additives via reduced amino acid density by 5% and ME by 1.5%; **(F)** Reduced diet + 0.15% Fibrafid; **(G)** Reduced diet + 0.25% Fibrafid; **(H)** Reduced diet + 0.10% TURBO Grow. As illustrated in this figure, histological evaluation of jejunum tissues at day 35 demonstrated intact villi and Lieberkühn’s intestinal crypts architecture, and mucosal layers in all treated groups. No signs of hyperplasia or dysplasia were observed, indicating healthy cell regeneration and absorb nutrients normally. The absence of inflammation or tissue damage highlights Fibrafid’s role in maintaining gut health, even under nutrient-reduced diets. Magnification under object lens 100x and scale bar 100 μm.

As illustrated in Figures 3A–H, histological evaluation of jejunum tissues at day 35 under HS demonstrated normal architectures of villi and crypt in all treated groups. Lieberkühn’s intestinal crypts are located below the villi, which are normal crypts without hyperplasia (excessive growth) or dysplasia (abnormal growth) involved in cell renewal. Simple columnar epithelial cells with goblet cells can be seen secreting mucus to protect the lining and allow smooth passage of food. The crypts underlying the villi are intact and show no significant pathological changes, indicating normal cell regeneration. The mucosal layers appear to be intact and show no visible inflammation, erosion or tissue damage.

## 4 Discussion

The present study demonstrates that both dietary nutrient density and additive supplementation significantly influenced growth performance, carcass traits, meat quality, and intestinal histopathology in heat-stressed broilers. Importantly, the factorial analysis clarified that most additive effects were consistent across both diet types, with limited but meaningful diet × additive interactions. A low-protein diet may help to manage the features of the fecal microbial population under hot temperature circumstances without negatively impacting broiler performance (29). This study evaluates the effects of these additives individually on the physiochemical properties of broiler breast meat under varying nutritional conditions.

Under HS, postmortem glycolysis is often accelerated, resulting in abnormal muscle pH that can manifest as PSE (pale, soft, exudative) or DFD (dark, firm, dry) meat-both undesirable for meat quality. In the present study, the pH of breast muscle at 24 h’ postmortem (PM24) was influenced by diet, with reduced diets showing slightly higher values, indicating a slower postmortem glycolysis compared with birds fed the standard diet. However, muscle pH remained within the normal physiological range (5.61–5.69) and was not altered by additive supplementation or by diet*additive interaction, indicating that additive effects were consistent across both diets. Thus, the pH values reflect normal postmortem glycolysis across all groups. A study by Garcia, De Freitas (7) observed that associated pH stability with reduced spoilage, glycolytic imbalance, and improved color traits. Color parameters were moderately influenced by both diet and additive, with several interaction effects. The enhanced lightness (L^*^) and redness (a^*^) in Fibrafid groups reflect improved myoglobin stability and reduced oxidation, both key indicators of freshness and consumer appeal. The reduced diet + 0.25% Fibrafid group showed the most consistent color stability despite dietary restrictions, underscoring the additive’s effectiveness under suboptimal nutritional conditions. These findings are in line with Akter, Kalam (30) and Wang, Xiao (31), who reported similar improvements in meat color and pH using green macroalgae and *Bacillus subtilis*, respectively.

WHC is a critical determinant of meat juiciness, texture, and yield. HS and nutrient-reduced diets are known to impair protein integrity, leading to increased drip loss and cooking loss (CL). In this study, Fibrafid (0.25%) significantly improved WHC and reduced both DL and CL, suggesting enhanced protein structure stability and reduced moisture migration. These findings support previous research indicating that natural feed additives can mitigate protein denaturation and preserve structural integrity (8, 9). The enhanced WHC is also associated with improved tenderness and juiciness, key sensory qualities valued by consumers. Similar reductions in moisture loss have been reported with dietary prebiotics and phytogenics (6, 10), reinforcing the role of Fibrafid in meat quality enhancement.

Tenderness is largely influenced by connective tissue, sarcomere length, and myofibrillar degradation. The observed increase in MFI in Fibrafid-treated groups suggests a higher rate of proteolysis and muscle softening, contributing to enhanced tenderness. TPA further validated this effect, showing higher springiness and cohesiveness, and lower hardness and chewiness in Fibrafid groups-particularly standard diet + 0.25% Fibrafid. Although Warner-Bratzler shear force (WBSF) values did not differ significantly among treatments (all ~3.0 kg), improvements in tenderness were evident from higher myofibrillar fragmentation index values and favorable texture profile parameters (springiness, cohesiveness, reduced hardness). Therefore, tenderness improvements may be attributed mainly to proteolytic activity reflected in MFI and TPA, rather than shear force. These improvements indicate that Fibrafid not only preserved structural integrity but also enhanced the mechanical properties of meat. Wang, Xiao (31) and Akter, Kalam (30) similarly reported enhanced tenderness with natural dietary additives, linked to shifts in muscle fiber composition and improved antioxidant status.

Carcass characteristics were broadly unaffected by diet or additive, although breast yield was consistently higher in Fibrafid-supplemented groups compared with controls, indicating improved nutrient partitioning toward high-value cuts. The heavier gizzards observed, especially in reduced diet + 0.25% Fibrafid, suggest enhanced mechanical digestion, which may support better feed processing and utilization. These findings are in agreement with Al-abdullatif, Al-Garadi (21), who observed increased breast yield with probiotic supplementation. Increased gizzard yield may reflect improved gut motility and enzyme activity, as also shown in studies on fiber-rich diets Liebl, Gierus (32). The enhanced thymus weights in treated groups further suggest immunomodulatory effects, likely due to improved gut-immune axis function. This is consistent with findings by Shehata, Yalçın (33) and Balcells, Martínez Monteros (34), who linked prebiotic and probiotic supplementation with thymic development and T-cell proliferation. Reduced diets, when supplemented with Fibrafid, produced yields comparable to standard diets, emphasizing the potential for nutrient sparing without compromising product quality.

Reduced-energy and amino acid density diets are typically associated with impaired growth and FCR. However, Fibrafid supplementation significantly improved ABW and FCR, particularly in the 0.25% groups. These outcomes were evident during both thermoneutral and heat-stress phases, demonstrating Fibrafid’s capacity to mitigate the negative effects of environmental and nutritional stressors. This agrees with Akter, Kalam (30) and Zhao, Niu (35), who reported that plant-derived additives enhance gut health, feed efficiency, and growth performance. The improved nutrient digestibility likely stems from Fibrafid’s bioactive compounds, which may modulate OS and gut microbiota composition. Similarly, TURBO Grow-treated groups also showed improved performance, reinforcing the synergistic benefits of combining phytogenics and prebiotics (36, 37). In contrast, the reduced diet without additives group’s poor performance underscores the risks of nutrient-restricted diets without adequate supplementation. Although Fibrafid has been associated with antioxidant and gut-protective properties, the present study did not directly measure OS, immune parameters, or microbiota composition. Future studies incorporating biochemical markers, immunological, and microbiological assays are needed to substantiate the underlying pathways by which Fibrafid may exert its effects.

Maintaining gut integrity is critical under HS and dietary restrictions. Histopathological examination showed that the jejunum of Fibrafid-treated birds exhibited no signs of inflammation, structural damage, or developmental abnormalities, suggesting that Fibrafid is safe for long-term use. Thus, Fibrafid as a natural feed supplement can contribute to a better intestinal environment by promoting favorable conditions for nutrient absorption, even in a low calorie and low protein diet. These findings align with Bolvig, Adlercreutz (38) and Shalaby, Hassan (39), who reported improved intestinal architecture following phytogenic supplementation. Bioactive compounds such as resveratrol, thymol, and berberine have been shown to enhance tight junction function and reduce inflammatory signaling, contributing to improved gut barrier integrity and nutrient transport efficiency. In this study, jejunal cross-sections were evaluated qualitatively to identify histopathological alterations under HS. A limitation, however, is that villus height and crypt depth were not measured quantitatively. Future studies should therefore include detailed morphometric analysis to provide more definitive evidence.

Collectively, these findings indicate that Fibrafid supplementation, particularly at 0.25%, enhances both meat quality and feed conversion to gain efficiency under HS. The benefits were evident across diet types, underscoring its robustness as a feed additive. Therefore, these findings underline the importance of strategies that enhance feed efficiency under HS, as such improvements not only sustain productivity but also support broader goals of sustainability and food security in the face of global climate change.

## 5 Conclusion

In conclusion, Fibrafid supplementation improved breast yield and breast meat quality, final ABW and FCR in heat-stressed broilers, with the strongest effects observed at 0.25% inclusion. Benefits included higher WHC, lower CL, improved MFI, and keeper jejunal integrity. TURBO Grow supplementation showed intermediate benefits, serving as a valuable commercial comparator. These effects were consistent across both standard and reduced diets, with limited but meaningful diet × additive interactions. Meat pH and shear force remained unaffected by additive. Importantly, reduced diets supplemented with Fibrafid maintained carcass yields comparable to standard diets, supporting more sustainable feeding strategies. By mitigating heat stress effects, supporting production on lower-energy diets, optimizing nutrient utilization and reducing feed costs Fibrafid additives contribute to sustainable, profitable, and resilient poultry production systems. Additionally, its potential to mitigate OS and improve gut health aligns with industry efforts to reduce the environmental impact of poultry farming. To back up this claim, additional research is needed to assess Fibrafid’s potential economic and environmental benefits. In addition, future research should incorporate oxidative and immune markers to elucidate the mechanisms underlying these improvements.

## Data Availability

The original contributions presented in the study are included in the article/Supplementary material, further inquiries can be directed to the corresponding authors.
