# Supplementary material for: Effects of dietary Fibrafid as phytogenic supplementation in standard and nutrient-reduced diets on breast meat quality, carcass traits, histopathology, and feed efficiency in heat-stressed broilers
Source: Front Vet Sci. 2025 Oct 10;12:1671325. doi: 10.3389/fvets.2025.1671325 (PMC12551230; doi:10.3389/fvets.2025.1671325)
Supplement: Supplementary file 1 [file Table_1.docx]

**Supplementary Table**

Supplementary **Table S1.** Feed components and nutritional content of basal diets (%).

| Ingredients (%) | Broiler Starter (0-10 d) | | | | Broiler Grower (10-24 d) | | | | Broiler Finisher (24-35 d) | | | |
| --- | --- | --- | --- | --- | --- | --- | --- | --- | --- | --- | --- | --- |
|  | PC1 | PC2 | NC1 | NC2 | PC1 | PC2 | NC1 | NC2 | PC1 | PC2 | NC1 | NC2 |
| BAKERY MEAL | 2.52 | 2.16 | 2.4 | 2.37 | 1.9 | 1.87 | 2.58 | 2.47 | 10 | 10 | 3.29 | 3.18 |
| CORN | 53.7 | 53.95 | 56.53 | 56.49 | 58.22 | 58.18 | 61.51 | 61.55 | 57.43 | 57.41 | 64.87 | 64.91 |
| SBM 48% CP | 36.83 | 36.74 | 35.24 | 35.21 | 33.32 | 33.29 | 30.73 | 30.7 | 27.94 | 27.9 | 27.21 | 27.19 |
| WHEAT BRAN | 0.05 | 0.05 | 0.05 | 0.05 | 0.00 | 0.00 | 0.00 | 0.00 | 0.00 | 0.00 | 0.00 | 0.00 |
| SOY OIL | 2.89 | 2.89 | 1.8 | 1.8 | 3.32 | 3.32 | 2.00 | 2.00 | 2.08 | 2.07 | 2.00 | 2.00 |
| MCP | 1.00 | 1.00 | 1.00 | 1.00 | 0.67 | 0.67 | 0.68 | 0.68 | 0.44 | 0.44 | 0.45 | 0.45 |
| LIMESTONE | 1.3 | 1.3 | 1.3 | 1.3 | 0.85 | 0.85 | 0.86 | 0.84 | 0.69 | 0.67 | 0.70 | 0.68 |
| DL-METHIONINE | 0.33 | 0.42 | 0.38 | 0.38 | 0.37 | 0.37 | 0.33 | 0.33 | 0.32 | 0.32 | 0.31 | 0.31 |
| L-LYSINE HCL | 0.4 | 0.41 | 0.36 | 0.36 | 0.32 | 0.32 | 0.32 | 0.32 | 0.33 | 0.33 | 0.30 | 0.30 |
| L-THREONINE | 0.15 | 0.15 | 0.15 | 0.15 | 0.12 | 0.12 | 0.11 | 0.12 | 0.01 | 0.01 | 0.00 | 0.00 |
| L-VALINE | 0.10 | 0.10 | 0.10 | 0.1 | 0.07 | 0.07 | 0.06 | 0.06 | 0.06 | 0.06 | 0.05 | 0.05 |
| COMMON SALT | 0.32 | 0.32 | 0.29 | 0.29 | 0.32 | 0.33 | 0.31 | 0.32 | 0.19 | 0.19 | 0.30 | 0.30 |
| VITAMIN PREMIX | 0.10 | 0.10 | 0.10 | 0.10 | 0.10 | 0.10 | 0.10 | 0.10 | 0.10 | 0.10 | 0.10 | 0.10 |
| MINERAL PREMIX | 0.10 | 0.10 | 0.10 | 0.10 | 0.10 | 0.10 | 0.10 | 0.10 | 0.10 | 0.10 | 0.10 | 0.10 |
| T-BINDER | 0.10 | 0.10 | 0.10 | 0.10 | 0.10 | 0.10 | 0.10 | 0.10 | 0.10 | 0.10 | 0.10 | 0.10 |
| TURBO GROW | 0.00 | 0.10 | 0.00 | 0.10 | 0.00 | 0.10 | 0.00 | 0.10 | 0.00 | 0.10 | 0.00 | 0.10 |
| LIGNOBOND | 0.10 | 0.10 | 0.10 | 0.10 | 0.20 | 0.20 | 0.20 | 0.20 | 0.20 | 0.20 | 0.20 | 0.20 |
| OptiPhos® Plus | 0.01 | 0.01 | 0.01 | 0.01 | 0.01 | 0.01 | 0.01 | 0.01 | 0.01 | 0.01 | 0.01 | 0.01 |
| TOTAL | 100 | 100 | 100 | 100 | 100 | 100 | 100 | 100 | 100 | 100 | 100 | 100 |
| Calculated Composition |  |  |  |  |  |  |  |  |  |  |  |  |
| M.E. | 2975 | 2975 | 2930 | 2930 | 3050 | 3050 | 3004 | 3004 | 3100 | 3100 | 3054 | 3054 |
| Crude Protein | 23.08 | 23.07 | 22.43 | 22.43 | 21.50 | 21.49 | 20.58 | 20.58 | 19.88 | 19.88 | 19.16 | 19.16 |
| Ca | 0.95 | 0.95 | 0.95 | 0.95 | 0.75 | 0.75 | 0.75 | 0.75 | 0.65 | 0.65 | 0.65 | 0.65 |
| Avail. P | 0.50 | 0.50 | 0.50 | 0.50 | 0.42 | 0.42 | 0.42 | 0.42 | 0.36 | 0.36 | 0.36 | 0.36 |
| Na | 0.18 | 0.18 | 0.18 | 0.18 | 0.19 | 0.19 | 0.19 | 0.19 | 0.19 | 0.19 | 0.19 | 0.19 |
| dLYS | 1.32 | 1.32 | 1.25 | 1.25 | 1.18 | 1.18 | 1.12 | 1.12 | 1.08 | 1.08 | 1.03 | 1.03 |
| dMET | 0.72 | 0.72 | 0.67 | 0.67 | 0.65 | 0.65 | 0.61 | 0.61 | 0.61 | 0.61 | 0.57 | 0.57 |
| dTSAA | 1.00 | 1.00 | 0.95 | 0.95 | 0.92 | 0.92 | 0.87 | 0.87 | 0.86 | 0.86 | 0.82 | 0.82 |
| dTHR | 0.88 | 0.88 | 0.84 | 0.84 | 0.79 | 0.79 | 0.75 | 0.75 | 0.72 | 0.72 | 0.69 | 0.68 |
| dTRP | 0.27 | 0.27 | 0.26 | 0.26 | 0.25 | 0.25 | 0.23 | 0.23 | 0.23 | 0.23 | 0.22 | 0.22 |
| dARG | 1.37 | 1.37 | 1.33 | 1.33 | 1.27 | 1.27 | 1.21 | 1.21 | 1.16 | 1.16 | 1.11 | 1.11 |
| dVAL | 1.00 | 1.00 | 0.95 | 0.95 | 0.91 | 0.91 | 0.86 | 0.86 | 0.84 | 0.84 | 0.80 | 0.80 |
| Formula Cost (SR) | 1969 | 1980 | 1898 | 1909 | 1941 | 1952 | 1861 | 1872 | 1899 | 1910 | 1826 | 1838 |

PC1: positive control diet meeting the requirements of broiler chickens based on Ross Nutrient Specifications 2022 and containing no prebiotic (as TURBO Grow). PC2 = PC1 + TURBO Grow. NC1: Negative control diet 5% lower in amino acid density and 1.5% lower in ME and containing no prebiotic (as TURBO Grow). NC2 = NC1 + TURBO Grow.
